# Implicit acoustic sequence learning recruits the hippocampus

**DOI:** 10.1371/journal.pone.0209590

**Published:** 2018-12-21

**Authors:** Julia Jablonowski, Philipp Taesler, Qiufang Fu, Michael Rose

**Affiliations:** 1 NeuroImage Nord, Department for Systems Neuroscience, University Medical Center Hamburg Eppendorf, Martinistrasse, Hamburg, Germany; 2 State Key Laboratory of Brain and Cognitive Science, Institute of Psychology, Chinese Academy of Sciences, Beijing, China; Tokai University, JAPAN

## Abstract

The exclusive role of the medial temporal lobe in explicit memory has been questioned by several studies reporting medial temporal lobe involvement during implicit learning. Prior studies have demonstrated that hippocampal engagement is present during the implicit learning of perceptual associations, however, it is absent during learning response-related associations. Therefore, it was hypothesized that the function of the medial temporal lobe during implicit learning is related to the extraction of perceptual associations in general. While in most implicit learning tasks visual stimuli were used, the aim of the current functional magnetic resonance imaging (fMRI) study was to detect whether activations within medial temporal lobe structures are also found during implicit learning of auditory associations. In a modified version of the classical serial reaction time task, participants reacted to the presentation of five different tones. Unbeknownst to the participants, the tones were presented with an underlying sequential regularity that could be learned. To avoid an influence of response learning on acoustic associative learning, response buttons were remapped in every trial. After learning, two different tests were used to measure participants’ conscious knowledge about the underlying sequence in order to assess the amount of implicit memory and to exclude participants with explicit knowledge acquired during learning. fMRI results revealed hippocampal activations for implicit learning of the acoustic sequence. When detecting a relation between implicit learning of acoustic associations and hippocampal activations, this study indicated a relation between hippocampal activations and memory formation of perceptual-based relational representation regardless of explicit knowledge. Thus, present findings suggest a general functional role for the formation of sequenced perceptual associations independent of the involvement of awareness.

## Introduction

The acquisition and use of knowledge can occur incidentally and without the involvement of awareness. This phenomenon is described as implicit learning and was first introduced by Reber [1,2]. A prominent paradigm to investigate implicit sequence learning is the serial reaction time (SRT) task [3] in which participants learn to react to a fixed set of stimuli while being unaware of the existence of an underlying regularity. When assessing participants’ knowledge after learning, above chance performance and a lack of awareness concerning the underlying sequence suggest the acquisition of implicit sequence knowledge [3–5].

At the neural level, it remains controversial as to which neural structures can be associated with implicit sequence learning. Preliminary SRT investigations revealed the involvement of the basal ganglia and motor cortex regions during implicit sequence learning [6–10], which led to the assumption that the basal ganglia are associated with the implicit learning system. However, other studies using the classical version of the SRT task reported the recruitment of the hippocampus within the medial temporal lobe (MTL) during implicit learning [11–14]. A recent study even reported activations in the hippocampus for both implicit and explicit sequence learning [14]. This means, regardless of whether or not explicit knowledge was gained during the SRT task, activation in the hippocampus is reported, which is contrary to the pervious assumption that the MTL involvement is exclusively linked with conscious learning processes [15,16]. Furthermore, these studies challenge the assumption of whether the basal ganglia are exclusively related to implicit learning.

These controversial results can be attributed in part to different learning mechanisms that are employed during the SRT task. In the classical version of the SRT task, each target stimulus is mapped to a fixed button of a fixed button set. Since the perceptual and motor response sequences are structured identically, both sequences are perfectly correlated. Therefore, implicit learning can occur within the stimulus or motor modality, or within a combination of both modalities. Hence, within previous SRT studies, implicit learning could have not been attributed to either modality, which may account for the different results found during sequence learning experiments.

Concerning the question of what exactly has been learned, associative learning in the SRT task can be explained by different learning mechanisms, such as response-response (R-R), stimulus-response (S-R), response-stimulus (R-S), and stimulus-stimulus (S-S) associative learning [17–19]. Learning the fixed R-S pairs allow participants to predict the next stimulus following each response. During S-S learning, the relationships in the stimuli structure sequence are learned. Each stimulus is followed by a specific stimulus comprising the underlying deterministic sequence of perceptual associations independently of what motor responses are given. Therefore, to investigate the neural structures involved in classical SRT tasks a clear differentiation between the perceptual and motor learning system is required.

Using a modified version of the SRT task, a recent study detected the implicit sequence learning of visual associations independent of motor response mapping while decorrelating the perceptual and motor sequence [20]. The perceptual and the motor modality were independently manipulated by implementing a trial-by-trial remapping of response buttons: in each trial, the assignment of the stimulus to the response location changed allowing a separation of the two domains. A perceptual sequence was established by a systematic variation of target stimuli across trials, whereas the motor sequence relied on a systematic variation of button presses. Hence, one group of participants exclusively learned a perceptual sequence while reacting to a random sequence of motor responses, and the other group selectively learned a motor sequence. Activation in the hippocampus was exclusively related to visual sequence learning and not to motor sequence learning which, in contrast, recruited the involvement of basal ganglia and motor cortex regions [20]. It was concluded that hippocampal activation found during the SRT task might not depend on whether explicit or implicit representations were acquired [20,21]. Activation of the hippocampus might rather depend on the modality in which the sequence is processed and thus on which stimulus features the underlying sequence is based on. Due to the fact that previous SRT experiments have mainly used visual stimuli, it is not clear as to whether the functional role of the hippocampus can be generalized to perceptual sequence learning or whether it is specific to the visual domain.

Based on this study, the aim of the present study is to test the hypothesis whether the hippocampus is relevant for the generation of sequence knowledge in the auditory modality. The assumed generalized functional role for the implicit formation of sequenced perceptual representations would be supported if hippocampal effects can be related to implicit sequence learning of acoustic associations.

To investigate this, we use a modified version of the SRT task with a deterministic sequence of auditory stimuli. To exclude motor response learning, S-R mapping was rearranged for each trial. After completing the SRT task, the amount of implicit knowledge and possible explicit knowledge were estimated using a completion task in combination with a confidence rating, a free-generation test and a post-experimental interview. The completion task is a well-established method for measuring participants’ conscious states about their acquired knowledge [22–24]. During the completion task the participants were asked to predict the identity of the next stimulus after the presentation of one tone and to indicate their confidence about their decision. It was shown that explicit memory results in more accurate responses with a higher degree of confidence. While implicit memory also results in more accurate responses, correct responses were equally distributed between high and low confidence ratings.

## Methods

### Overview of the experiment

#### Participants

Overall, 16 healthy individuals volunteered (between 18–36 years old, 6 females) to participate in the present fMRI experiment. All participants were right handed and had normal or corrected-to-normal vision. The fMRI study was approved by the ethics committee of the “Deutsche Gesellschaft für Psychologie” (DGPs) and all participants provided informed consent before participating in the experiment.

#### Stimuli

Two black arrows were presented on a white square (0.6° x 0.6°; distance 2.3°) against a grey background screen. These arrows were directed either upwards or downwards on the white squares which correspond to the two response buttons, left and right. A computer was synchronized with the MR scanner using the “Presentation” software (http://www.neurobs.com/), which allowed for the manipulation of the experimental stimuli as well as the subsequent recording of data. Auditory stimuli consisted of five sinusoidal tones differing in frequency (400 Hz, 600 Hz, 800 Hz, 1200 Hz, and 1800 Hz) which were presented to participants via MR-compatible headphones.

For the fMRI experiment, an LCD projector was used to display the stimuli on a screen positioned on top of the head coil, which was viewed by the participants using a mirror (10 x 15° field of view). Participants’ responses were delivered using button presses on one of the two MR-compatible devices (one button for each hand).

#### Design

The whole experiment was subdivided into two parts: (1) a learning phase (SRT task), and (2) a test phase which was further subdivided into the completion task in combination with a confidence rating, the free-generation test, and the post-experimental interview. All participants performed the SRT task and the completion task within the MR device, followed by the free-generation test, the post-experimental interview and the debriefing outside of the scanner.

#### Learning phase

**Fig 1:**
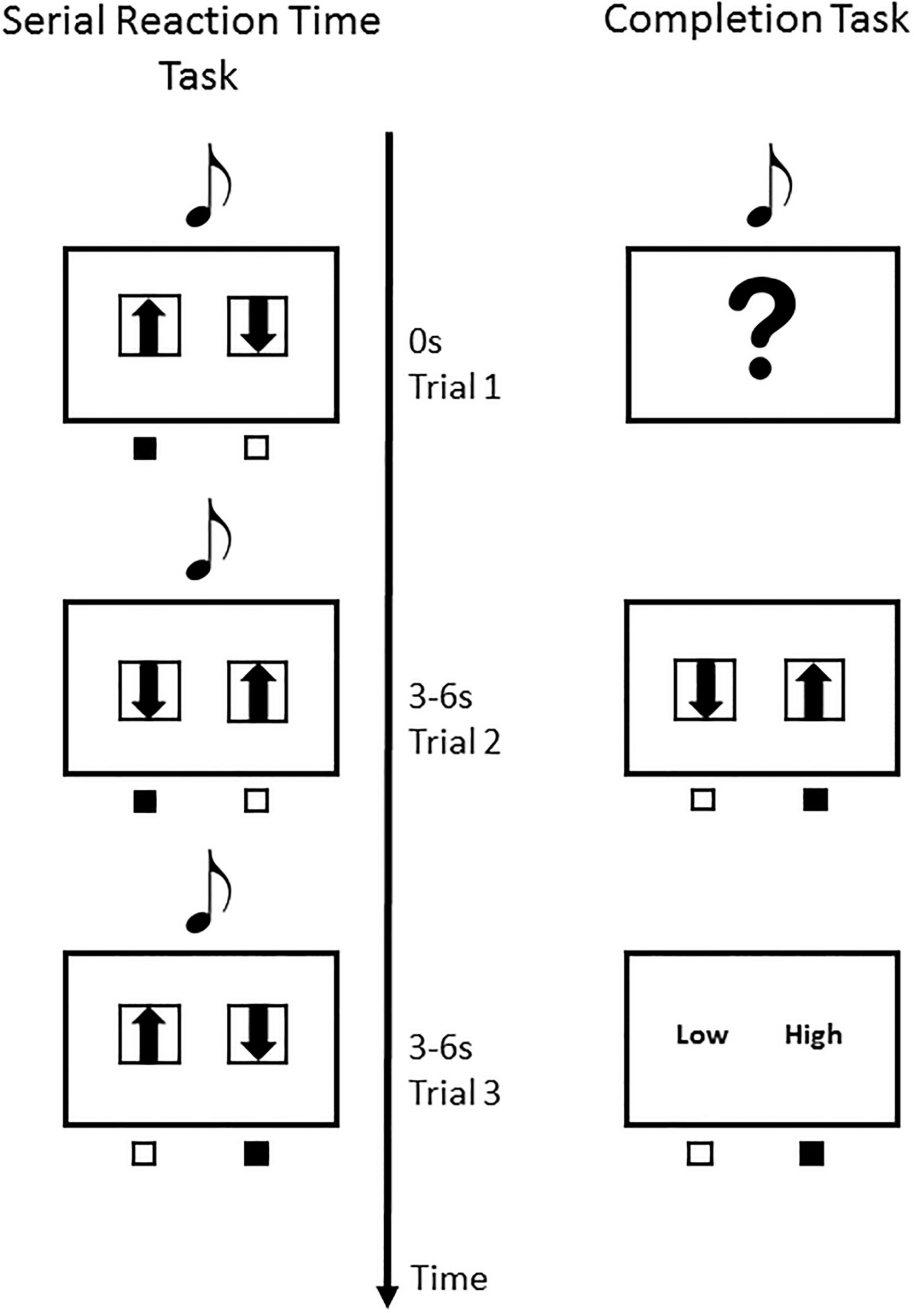
Example of the acoustic SRT task (left) and completion task (right). In both tasks, a sinusoidal tone was presented and responses were given via button press. The locations of the arrows, indicating the two response options (high vs. low), were assigned to corresponding buttons. In each trial of the SRT task, a remapping of the stimulus to the response was implemented to prevent motor learning. In the completion task, in contrast, after each tone presentation participants were instructed to predict the next tone as well as to rate their confidence by pressing the corresponding button.

The SRT task comprised of three sessions with 120 trials each. Breaks were included between sessions. During each trial, two arrows, pointing either upwards or downwards, and a sinusoidal tone was simultaneously presented to the participant. Due to scanner noise, the level of sound pressure was individually calibrated to be within the participant’s comfortable hearing level (approximately 80 dB). The tone lasted for 1 second, but the visual stimuli remained on the screen until a response was provided. The task was to identify whether the current tone was higher or lower than the previous tone. The arrows indicated the two response possibilities concerning the pitch rating (“higher tone” or “lower tone”) of the current tone (Fig 1). Responses were given by button presses: if the current tone was rated as being higher, the button corresponding to the arrow directing “upwards” should have been pressed. If the current tone was rated as being lower, the button corresponding to the arrow directing “downwards” should have been pressed. To prevent motor sequence learning the arrows were randomly arranged in each trial. However, the presentation of the tones was determined by a fixed stimulus sequence (tones: 4, 3, 1, 5, 2; this means: 1200 Hz, 800 Hz, 400 Hz, 1800 Hz, and 600 Hz). The participants were not informed about the order of the sequence in order to allow for the acoustic sequence to be learned incidentally, thus forming an implicit memory. Participants had to provide their responses within a window of 3 seconds. The length of the inter-trial interval varied between 3 and 6 seconds.

#### Test phase

The test phase consisted of two parts, the completion task in combination with a confidence rating, and the free-generation test in combination with a post-experimental interview. The completion task was used to identify the amount of implicit and explicit sequence knowledge acquired during the learning phase. This task was restricted to 20 trials and was identical to the SRT task except that after the presentation of each tone the sequence paused and a question mark was presented in the center of the screen (Fig 1). Participants were instructed to predict the next response by pressing the corresponding response button: if they predicted the next tone to be lower, they had to press the left button; if they predicted the next tone to be higher, they had to press the right button. Each tone was presented 4 times, and in the same presentation sequence as in the SRT task. When a response was given, the task continued with a confidence rating concerning the correctness of their response. This was performed by pressing one of the corresponding response button: if the response was made with low confidence, they were instructed to press the left button which was associated with the rating “I am guessing”; if the response was made with high confidence, they were instructed to press the right button, which was associated with the rating “I am sure”. Across participants, above chance performances and no significant difference between high and low correct responses were used as one indicator for participants’ implicit sequence memory.

Outside of the scanner, participants performed a free-generation test in combination with a post-experimental interview. Participants were asked whether they have noticed anything special during the experiment. If not, they were directly asked whether they have noticed an underlying regularity. If so, participants were asked to verbally report and draw the underlying regularity onto paper. This allowed us to rule out the participants which demonstrated explicit knowledge, and to ensure that no included participants were aware of the presence of any kind of regularity. In the free-generation test, all participants were asked to produce a sequence of tones, consisting of 35 elements, which resembled the sequence within SRT task as much as possible. This was done using five consecutive buttons on the keyboard which represented the tones from the lowest to the highest pitch, respectively. Participants’ performance on the free-generation test was examined by calculating the amount of correctly recalled tone-to-tone transitions. Hence, participants which might have explicit knowledge or partial explicit knowledge but lacked confidence to verbally report it could also be detected. In summary, the completion task, the verbal report, and the free-generation test were used as criteria to exclude participants with explicit memory.

### Behavioral data analysis

For the behavioral analysis of the learning phase of the SRT task, we calculated the amount of incorrect responses, for each participant. We excluded participants who indicated a high level of errors (> 30% errors).

For the behavioral analysis of included participants, the trials with incorrect or extraordinary slow responses, characterized by a latency above 2000 milliseconds, were excluded from further behavioral and fMRI analyses. Further, we calculated and reported the effect sizes of the behavioral analyses using Cohen’s *d* (*d*) and eta squared (η2) [25,26].

For the reaction time analysis, we calculated the mean reaction time over time across the learning phase of the SRT task with respect to the onset of the correct response for each single input.

For the test phase, the completion task performance was used to exclude participants with explicit memory and to assess the amount of acquired implicit knowledge. The completion task performance was grouped into correct and false responses, whereas correct responses were further subdivided into “high confidence” or “low confidence” responses. This classification contributed to differentiate between explicit (high amount of correct responses and high confidence ratings) and implicit (high amount of correct responses and equal distribution between high and low confidence ratings) knowledge, and hence to exclude individuals indicating explicit knowledge. For the participants with implicit knowledge, we calculated the amount of correct responses in percentage of each tone-to-tone transition, referred to as the completion task score. We used the completion task score as a measure for the amount of implicit sequence knowledge acquired in the learning phase of the SRT task. This score was used in later neuroimaging analysis in order to relate the acquired implicit knowledge separately for each tone-tone transitions to the tone-specific neural responses during the last session of the learning phase.

The free-generation test and the postexperimental questionnaire were particularly used to detect participants with explicit knowledge. Participants’ performance on the free-generation test was examined by calculating the amount of correctly recalled tone-to-tone. Participants were excluded if the acquired knowledge concerning the underlying sequence was correctly transferred to the free-generation task or if the sequence was verbally reported during the postexperimental interview.

### fMRI parameters and imaging analysis

Imaging was performed at the University Hospital of Eppendorf in Hamburg using a 3 T MR Scanner (Siemens Trio) with a standard gradient echo-planar imaging T2*-sensitive sequence with 36 contiguous axial slices (2mm thickness with 1mm gap, repetition time (TR) 2.178 s, echo time (TE) 25ms, flip angle 80°, field of view 216 mm^2^). A high-resolution (1x1x1 mm voxel size) structural MRI was acquired for each participant using a standard three-dimensional T_1_-weighted FLASH sequence.

Image processing and statistical analysis were carried out using SPM12 (http://www.fil.ion.ucl.ac.uk/spm/). Volumes were realigned to the middle volume, spatially normalized to a standard EPI template image of the MNI (Montreal Neurological Institute), and smoothed using a 6 mm full width at half maximum (FWHM) isotropic Gaussian kernel. The fMRI data were analyzed by an estimation of the BOLD signal for each tones (tone 1–5) and error response trials across the learning phase (learning session 1–3) convolved with a hemodynamic response function. A high-pass filter with a cut-off period of 120 s and a low-pass filter (Gaussian envelope FWHM of 4 s) were used. Using a general linear model, regression coefficients were estimated for each regressor. We performed a contrast for each tone-to-tone transition and transmitted the contrast images to the second level.

For the second level analysis, we used a flexible factorial design with inter-subject variability as random effects. The model included one factor for the subject factor and five factors for each tone. As a parameter for the acquired implicit memory, we included the completion task score as a covariate within the second level model for each tone and each subject, respectively.

Finally, we tested the effect of the covariate in order to investigate the different modulations of BOLD responses in relation to the acquired implicit memory. Due to the fact that only one completion score for each tone in one subject can be computed, the inclusion of a covariate at the first level was not possible. Hence, the effect of the covariate could have only be estimated at the second level across subjects. Relating participants’ acquired implicit knowledge to the neural responses of the last SRT session, we were able to examine the incidental learning effect.

For group analyses, the cluster-defining threshold adopted was p < 0.001. According to our a priori hypothesis, the hippocampus was defined as a Region of Interests (ROI). We centered the volume of interest on the coordinates according to a previous study by Rose et al. (2011) [20] on implicit learning of a pure perceptual sequence (left: x = -34, y = -20, z = -18; right: x = 28, y = -24, z = -18). For the a priori ROI analysis we corrected for multiple comparison (FWE threshold at p < 0.05) based on a search volume of 600mm^3^.

## Behavioral results

### Serial reaction time task

One participant indicating a high level of errors (>30% errors) was excluded from further analysis. Incorrect and extraordinary slow responses, characterized by a latency above 2000 milliseconds, were excluded from further behavioral and fMRI analyses. Across participant, an average 9% of all trials were excluded from analysis.

After learning, we excluded 4 participants indicating explicit knowledge assessed by using the three different criteria: the completion task, the free-generation test and the participants’ verbal report. Accuracy was calculated as the mean error rate per session. Overall, accuracy was high (Mean 93%).

For reaction time analysis, the mean reaction time was calculated with respect to the onset of the correct response for each single input. A repeated measures ANOVA with the within subject factor session revealed a significant decrease in reaction time over time (mean: session 1 = 888.5 ms; session 2 = 792.4 ms; session 3 = 804.4 ms; factor session: F(2,36) = 12.61, p < .001, η2 = 0.38).

### Completion task

Within the group of implicit participants, the completion task performance (percent correct trials), we found a significant difference between the amount of correct and incorrect trials (mean score: correct = 13.75 (68.75%); incorrect = 6.25 (31.2%); T(11) = 3.45, p = .005, *d* = 2.18) indicating acquired sequence knowledge after the learning phase of the SRT task. Among correct responses, there was no difference between high and low confidence trials (T(11) = 1.84, p = .09, *d* = 1.11), hence participants’ performance relied on implicit knowledge.

Although none of the included participants noticed any regularity, the tone with the highest pitch attracted participants’ attention significantly more than others during the SRT task, and was reported afterwards as especially noticeable and unpleasant. Due to the different level of attention and the unpleasantness of this tone, related trials were excluded from further analyses. Hence, we then tested whether there is a significant difference between the amount of correct and incorrect trials when excluding the salient tone from analysis.

**Fig 2:**
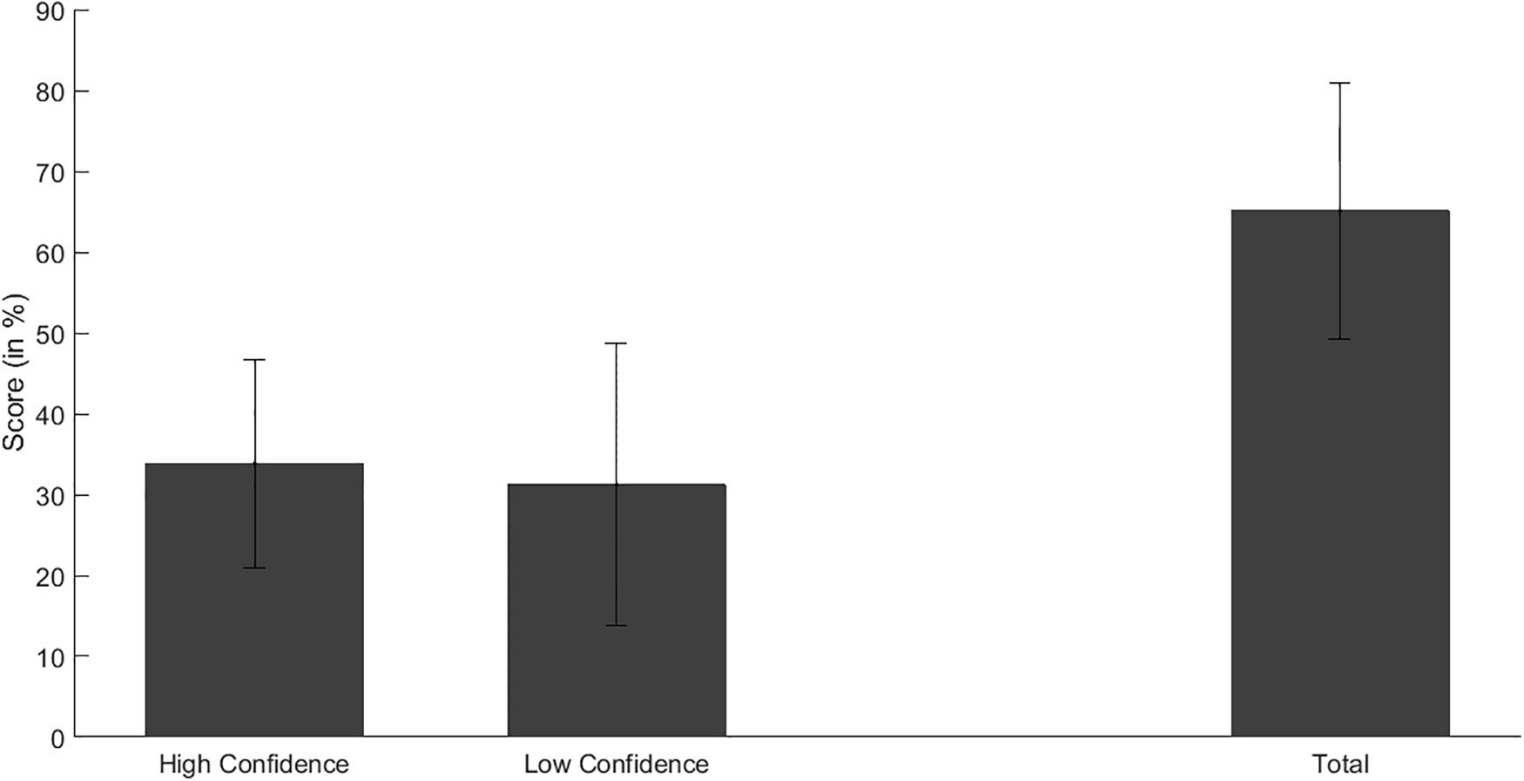
Behavioral results. Within the group of implicit participants, mean completion task performance (in percentage) for correct responses is shown: correct responses with high confidence, correct responses with low confidence, and total amount of correct responses (from left to right). Error bars denote the standard deviation. No significant difference between high and low confidence scores was found.

Within the group of implicit participants, the completion task performance (correct trials in percent), the significant difference between the amount of correct and incorrect trials remained (mean score: correct = 10.41 (65.1%); incorrect = 5.58 (34.9%); T(11) = 3.3, p = .007, *d* = 1.99) when excluding the salient tone from analysis. Further, to ensure reliable implicit sequence knowledge, we used a one sample t-test to test whether the summed value (65%) of the completion task performance (correct trials in percent) is above the chance level of 50% (T(11) = 3.29; p < .01, *d* = 1.99). Participants’ completion task performance was above chance level indicating that participants had reliable implicit knowledge about the sequential dependencies. Among correct trials, no difference between high and low confidence ratings was found (T(11) = 1.51, p = .16) (Fig 2). Since an equal distribution between high and low confidence ratings remained even when the salient tone was excluded from analysis, explicit knowledge cannot account for participants’ above chance SRT task performances and related sequence knowledge.

### Free-generation test

Within the group of implicit participants, performances on the free-generation test were evaluated by calculating the amount of correct recalled tone-to-tone transitions. None of the participants were able to recall neither the whole sequence, nor a portion of the underlying sequence, at an above chance level. Further, participants did not noticed any regularity during the SRT task or test session and hence were not able to verbally report the underlying sequence.

### Functional neuroimaging results

Throughout learning, participants acquired implicit sequence knowledge concerning some specific tones compared to the others. For the functional MRI analyses, we tested the individual relation between the BOLD signal change and implicit sequence knowledge acquired during the learning phase of the SRT task. For each tone, an individual completion task score was calculated and the completion task scores were included as a covariate for each participant.

**Fig 3:**
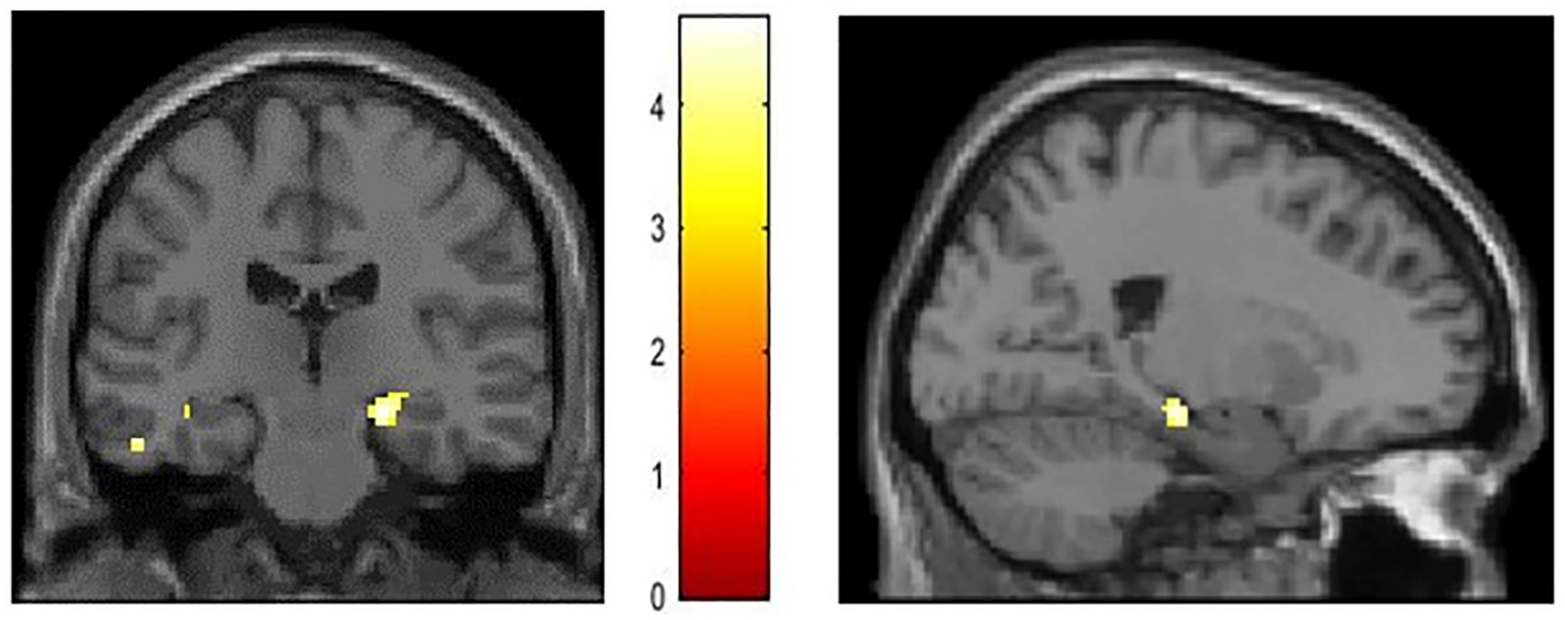
Statistical map thresholded at p < .001. Main effects of covariate show memory specific effects. Results indicate a positive relation between the amount of acquired knowledge (completion task scores) and significant effects within voxels in the right (-38, -22, -14) and left hippocampus (24, -22, -14) within the group of implicit participants (p < .05; FWE corrected).

Present results indicate a positive relation between the amount of acquired implicit sequence knowledge (completion task scores) and significant effects within voxels in the left hippocampus across participants (T(43) = 5.56, p = 0.001, at -38, -22, -12 [x y z]). When excluding the salient tone from analyses, a significant main effects of covariate indicated memory specific effects. A positive relation between BOLD signal changes within the left and right hippocampus (left: T(32) = 3.52, p = 0.001, at -38, -22, -14 [x y z], right: T(32) = 4.03, p < 0.001, at 24, -22, -14 [x y z]) and the amount of acquired implicit sequence knowledge (completion task scores) was found (Fig 3).

**Fig 4:**
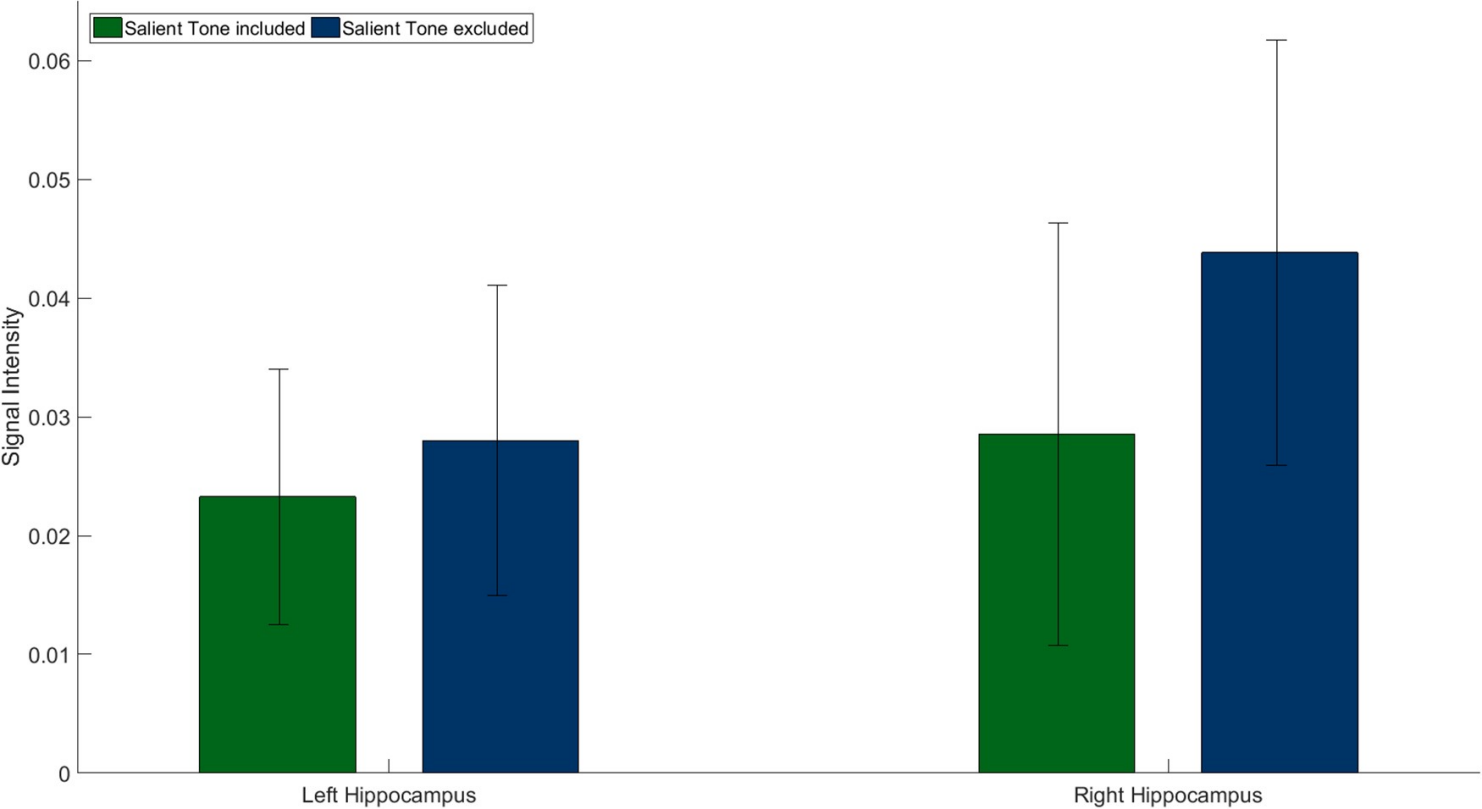
Effects of the memory specific covariate within ROI centered on coordinates of the left and the right hippocampus (contrast estimates and 90% confidence interval). A similar pattern of the memory specific covariate can be found in both analyses, when the salient tone is included (green; left: T(43) = 5.56, p = 0.001; right: n.s. after correction for multiple comparison) and excluded (blue; left: T(32) = 3.52, p = 0.001; right: T(32) = 4.03, p < 0.001) from analysis. However, the effect of the memory specific covariate is enhanced when the salient tone is excluded.

For a comparison of the memory specific covariate effects within ROI of both analyses, when the salient tone was included and excluded, we compared the contrast estimates between both analyses in the left and right hippocampus. A similar pattern of the memory specific covariate effects was observed in both analyses (Fig 4). However, the effect of the memory specific covariate was enhanced when the salient tone had been excluded. We thus concluded that the salient tone evoked different cognitive processes than the other tones, which was also reflected in the BOLD signal changes in the hippocampus.

## Discussion

In this study, we provide evidence for a functional role of the hippocampus in implicit acoustic sequence learning. We observed a positive relation between the BOLD signal changes within the left and right hippocampus and the amount of acquired sequence knowledge without the generation of explicit memory. In accordance with a previous study [20], these findings suggest a general functional role of the hippocampus within MTL structures in implicit sequence learning of perceptual associations.

In the present study, implicit sequence learning was assessed by using a modified acoustic version of the SRT task in which participants learned a fixed sequence of perceptual stimuli while the acquired knowledge was unconsciously generated. Decorrelating the stimulus sequence from the motor responses enabled us to avoid motor response learning and to explore the nature of implicit sequence learning of auditory associations independently. By the use of three different assessments, participants indicating any sign of explicit knowledge were excluded. Within the group of implicit participants, above chance performance in the completion task has been related to implicit memory for the perceptual sequence.

The present observation of implicit perceptual-based sequence learning is in line with previous behavioral SRT results [7,18,19,27–34]. Further, the effects in bilateral hippocampal structures were related to the amount of implicit memory for perceptual associations. In previous neuroimaging studies, some results suggested no involvement of the MTL in implicit sequence learning but the involvement of basal ganglia and motor cortex regions [6–9], while present and other studies revealed the recruitment of the MTL for implicit memory formation [11,13,20,21,35]. Further, some accounts exclusively related the involvement of MTL structures to sequence learning when explicit memory was involved [15,16].

The function of the MTL, in particular the hippocampus, has been attributed to memory and related to associative learning processes involving the integration of multiple stimulus events into relational representations [16,36–40]. This assumed function is in line with present findings indicating that the formation of associations between acoustic stimuli is mediated by hippocampal structures. In contrast to episodic memory, in the present study and also other studies, which used visual material, acquired knowledge was not accessible to awareness and therefore indicated implicit memory [14,20]. The formation of implicit relational representations in relation to MTL structure can also be observed for more complex material that requires the extraction of an abstract hidden rule in visual material [21,35]. The results of these studies indicated that the hippocampus mediated the flexible binding of temporally and spatially structured information into sequential representations according to their relationship.

On the level of single neuron recordings, a recent study provided further evidence for a fundamental role of the hippocampus during the flexible integration of spatial and nonspatial information into organized representations [36]. This study examined the rate and temporal coding of hippocampal neurons in rats while performing a task in which sounds and odor stimuli had to be sequentially integrated. While it is already known that the behavior of hippocampal place cells is associated with the memory formation of cognitive map representations, this study found that hippocampal event cells contribute to the temporal coding of episodic events. They concluded that the dynamic integration of spatial and temporal sequence information is controlled by the rate and temporal coding of event and place cells of hippocampal structures. Furthermore, this study introduced a new framework towards a more general model of the fundamental role of the hippocampus concerning the memory formation of episodic memory and spatial navigation.

Findings of previous SRT studies has not shown yet whether the implicit skill acquisition process is exclusively based on practicing a fixed S-R association or a fixed S-S association. The common issue among these implicit learning studies is that the question remains unclear whether the acquired implicit knowledge occurred within the stimulus or motor modality, or even within a combination of both modalities. Therefore, it is difficult to relate previous findings of neural correlates exclusively to implicit memory formation of relational perceptual associations. Consequently, in the present study the perceptual sequence was decorrelated from motor responses in order to relate findings of neural correlates exclusively to pure perceptual learning.

Using the same approach, a previous MR study used a modified visual version of the SRT task in order to examine visual sequence learning [20]. While investigating implicit learning effects of perceptual and motor associations independently, they found that the basal ganglia and motor cortex are involved in implicit motor sequence learning. In contrast, the hippocampus has been shown to be exclusively involved in implicit sequence learning of perceptual associations. It was suggested that different brain activation pattern are specific for the process of implicit sequence learning of perceptual or motor associations. Results from this study, using visual associations, as well as results from the current study, using acoustic associations, support the assumption that the functional role of the hippocampus during associative learning is determined by the material which is learned in the SRT task.

While using a different task, the serial color matching task, a recent neuroimaging study also examined learning-related neural correlates specific for implicit sequence information in the perceptual and motor domain [12,37]. While implicit learning effects were found for both the perceptual and the motor sequence, the effects of the hippocampus were less pronounced in the perceptual task compared to the motor task. The unexpected difference was explained by the fact that the motor learning task required the integration of a larger amount of information from different modalities. The results are in agreement with the present study and related assumption that the implicit binding process between perceptual associations recruits the involvement of the hippocampus.

A study conducted by Henke et al. (2003) investigated the neural correlates of implicit memory in general while not considering sequential material within their experimental design [13]. They examined the nonconscious encoding and retrieval of masked face-profession associations and related neural correlates while presenting stimuli within a visual masking paradigm. The masked presentations of faces and written profession pairs prevented the formation of explicit memory and hence allowed for the examination of neural correlates related to implicit associative learning. Using a forces-choice task, participants had to guess which profession belonged to which face revealing associative learning of implicit semantic stimuli pairs. The unconscious retrieval of the face-profession associations was related to hippocampal activation. In comparison to previous implicit sequence learning studies, this study further reveals that sequential material does not appear to be a mandatory factor for the involvement of the hippocampus.

While replicating important aspects of the study conducted by Henke et al. (2003), Degonda et al. (2005) also showed that implicit S-S associative learning is related to hippocampal activation [11]. In accord with our findings, these studies showed that the hippocampus is involved in the implicit memory formation of perceptual associations. Furthermore, both results contribute to the assumption that the involvement of the hippocampus in implicit learning has an important role in both sequential perceptual and non-sequential perceptual material.

Research into memory performances of amnesic patients provided a deeper insight into cognitive memory impairments resulting from damages to MTL structures. Using the SRT task, amnesic patients revealed impairments in implicit associative learning suggesting a relation between MTL activation and implicit sequence learning [38]. Amnestic patients with an extensive damage to the MTL performing a visual search task showed impaired performances in implicit learning of contextual information. While implicit perceptual skill learning was intact, patients revealed severe impairments in implicit contextual learning. Hence, the medial temporal memory system was related to implicit learning of contextual information which includes the binding of multiple cues [39,40]. These findings supports the relevance of the MTL involvement in implicit memory formation as observed in the present study.

A common factor of the studies mentioned above is that the acquired knowledge was implicitly extracted from perceptual input and MTL activation was reported during implicit learning. Noteworthy, previous neuroimaging SRT studies used visual stimuli as perceptual input. Suggesting a generalized functional role of the hippocampus for perceptual associative learning, however, requires the investigation of pure perceptual implicit learning using stimuli of a different modality than the visual. The current study used auditory stimuli in order to provide further evidence for implicit sequence learning of pure perceptual associations. While reporting a relation between hippocampal activations and implicit sequence learning of pure perceptual associations, current findings agree with previous reports. Therefore, we propose a generalized functional role of the hippocampus for the implicit formation of perceptual-based relational representation. With regard to the different learning systems involved in the different versions of the SRT task, the controversial findings we have mentioned previously can now be explained in more detail [12,14,20]. When investigating the implicit character within the motor domain, activations in basal ganglia and motor cortex regions are expected. In contrast, investigations into the implicit learning system of pure perceptual associations revealed that hippocampal activation contributed to the flexible integration of stimulus associations into an organized stimuli representation [41–46]. Present and previous findings hence challenge the traditional assumption that person’s level of awareness is mandatory for the involvement of the hippocampus within the MTL during associative learning [11–14,20,39,47–50].

Furthermore, dissociating the memory systems into whether associative learning occurred in the perceptual or in the motor domain is also in line with the findings on anatomical connections between sensory cortical pathways and MTL structures. Investigations into the anatomical connections between sensory cortical pathways and the MTL revealed that the hippocampus within MTL structures is perfectly suited to establish associations between perceptual stimuli [42,51,52]. Since the hippocampal formation receives unimodal and multi-modal input from all cortical association areas, present findings agree that sensory percepts are processed in the hippocampus.

Suggesting a generalized functional role of the hippocampus for implicit learning of pure perceptual relational representations is in line with a recent model addressing the role of the hippocampus in implicit learning processes [53]. Within this model, memory systems are not differentiated on the level of consciousness involvement, as in the traditionally manner, but on the level of functional contribution. It is assumed that the function of the hippocampus particularly concerns the rapid encoding of flexible associations independent of the involvement of explicit or implicit memory. Memory systems are instead differentiated by the stimuli modality depending on “what was learned”, and not by the involvement of participants’ awareness [20,30]. While the assumption that memory formation and hippocampal involvement regardless of the explicit character is in agreement with current data, present results further suggest that the hippocampus is involved not only during faster but also during slower implicit learning processes of pure perceptual-based associations. The present results clearly support the assumption that the memory system should be dissociated based on the properties of the learned material.

In summary, the present study investigated a relation of implicit auditory sequence learning and activations within hippocampal structures. In contrast to previous studies, we showed implicit perceptual learning effects using auditory sequence learning. While relating our findings with previous results on implicit memory formation, we suggest a generalized functional role of the hippocampus within MTL structures for the formation of perceptual relational representations independent of the involvement of awareness.
